# Unraveling the Anti-Cancer Mechanisms of Antibiotics: Current Insights, Controversies, and Future Perspectives

**DOI:** 10.3390/antibiotics14010009

**Published:** 2024-12-25

**Authors:** Nikolaos Nektarios Karamanolis, Dimitris Kounatidis, Natalia G. Vallianou, Krystalia Dimitriou, Eleni Tsaroucha, Georgios Tsioulos, Ioanna A. Anastasiou, Evangelos Mavrothalassitis, Irene Karampela, Maria Dalamaga

**Affiliations:** 1 Second Department of Internal Medicine, Hippokratio General Hospital, Medical School, National and Kapodistrian University of Athens, 11527 Athens, Greece; inektkaramanolis@gmail.com (N.N.K.); krystalia_dim@hotmail.com (K.D.); 2 Diabetes Center, First Department of Propaedeutic Internal Medicine, Laiko General Hospital, Medical School, National and Kapodistrian University of Athens, 11527 Athens, Greece; dimitriskounatidis82@outlook.com (D.K.); anastasiouiwanna@gmail.com (I.A.A.); 3 First Department of Internal Medicine, Sismanogleio General Hospital, 15126 Athens, Greece; natalia.vallianou@hotmail.com (N.G.V.); elenatsaroucha1@gmail.com (E.T.); vagsitis@gmail.com (E.M.); 4 Fourth Department of Internal Medicine, Attikon General University Hospital, Medical School, National and Kapodistrian University of Athens, 12462 Athens, Greece; geotsioulos@med.uoa.gr; 5 Second Department of Critical Care, Attikon General University Hospital, Medical School, National and Kapodistrian University of Athens, 12461 Athens, Greece; eikaras1@gmail.com; 6 Department of Biological Chemistry, Medical School, National and Kapodistrian University of Athens, 11527 Athens, Greece

**Keywords:** antibiotics, cancer, derivates, gut microbiota, immunotherapy, macrolides, quinolones, tetracyclines

## Abstract

Cancer persists as a significant global health challenge, claiming millions of lives annually despite remarkable strides in therapeutic innovation. Challenges such as drug resistance, toxicity, and suboptimal efficacy underscore the need for novel treatment paradigms. In this context, the repurposing of antibiotics as anti-cancer agents has emerged as an attractive prospect for investigation. Diverse classes of antibiotics have exhibited promising anti-cancer properties in both in vitro and in vivo studies. These mechanisms include the induction of apoptosis and cell cycle arrest, generation of reactive oxygen species, and inhibition of key regulators of cell proliferation and migration. Additional effects involve the disruption of angiogenesis and modulation of pivotal processes such as inflammation, immune response, mitochondrial dynamics, ferroptosis, and autophagy. Furthermore, antibiotics have demonstrated the potential to enhance the efficacy of conventional modalities like chemotherapy and radiotherapy, while alleviating treatment-induced toxicities. Nevertheless, the integration of antibiotics into oncological applications remains contentious, with concerns centered on their disruption of gut microbiota, interference with immunotherapeutic strategies, contribution to microbial resistance, and potential association with tumorigenesis. This narrative review explores the mechanisms of antibiotics’ anti-cancer activity, addresses controversies about their dual role in cancer biology, and envisions future perspectives that include the development of novel derivatives and innovative frameworks for their incorporation into cancer treatment paradigms.

## 1. Introduction

Cancer remains one of the most formidable global health challenges, causing millions of deaths annually and significantly impacting quality of life (QoL) and socioeconomic stability. Nearly one in five individuals aged 0–74 years will face a cancer diagnosis, with the prevalence of both new cases and associated mortality steadily climbing. In 2018, approximately 18 million new cancer cases were reported worldwide, a figure that rose to nearly 20 million in 2020, accompanied by 9.7 million cancer-related deaths. Projections for 2040 indicate a further surge, with an estimated 29.9 million new diagnoses and 15.3 million deaths attributed to cancer [1,2]. Among the various cancer types, lung and breast cancers remain predominant, while others, such as colorectal cancer (CRC), are becoming increasingly common [3].

Recent advances in oncology have deepened our understanding of the molecular and pathogenetic mechanisms underpinning cancer, laying the groundwork for novel therapeutic strategies. These mechanisms span several key biological pathways critical to tumor initiation and progression, including dysregulation of apoptosis, activation of oncogenic signaling pathways, and tumor-induced angiogenesis. Different signaling pathways, such as phosphatidylinositol-3 kinase/protein kinase B (PI3/Akt), the mammalian target of rapamycin (mTOR), and Wnt/β-catenin are intricately linked to cancer-related conditions such as altered mitochondrial function, increased production of reactive oxygen species (ROS), and metabolic reprogramming. These changes not only support rapid tumor growth but also create a microenvironment conducive to immune evasion and resistance to therapy [4,5]. An additional hallmark of cancer is the immune system’s failure to recognize and eliminate malignant cells, which has spurred significant progress in immunotherapies [3,6]. Despite these advances, the translation of cancer biology insights into effective therapies is fraught with challenges, including the high cost of drug development, complexities in clinical trial design, and the rigorous processes required to ensure drug safety.

In this context, drug repurposing—or identifying new therapeutic applications for existing drugs—has emerged as a promising strategy to expedite and economize drug development. An interesting example of research focusing on drug repurposing is provided by Corsello et al., who utilized the molecular barcoding method PRISM (profiling relative inhibition simultaneously in mixtures) and identified a plethora of non-oncology drugs that demonstrated selective anti-cancer action [7]. Additionally, Artificial Intelligence (AI) is predicted to aid in the repositioning of old drugs in cancer treatment, mitigating the need for extensive preclinical screening and chance observations to kickstart the process. Valuable modalities include the development of algorithms that anticipate interactions between compounds and specific cellular metabolic pathways or disease states [8].

Among the most intriguing candidates for repurposing are antibiotics, which have demonstrated unexpected yet significant anti-cancer potential in experimental settings. Beyond their traditional role in combating infections, several antibiotics have shown the ability to inhibit cancer cell growth, induce apoptosis, and interfere with processes critical to tumor progression, such as angiogenesis and metastasis. Their known safety profiles and well-characterized pharmacology make antibiotics particularly attractive for repurposing efforts. Advances in machine learning have further bolstered this strategy, offering innovative tools to identify and validate candidates for repurposing, including antibiotics with overlooked or novel anti-tumor properties [3,9].

Despite their potential, the use of antibiotics in oncology remains a part of an emerging and exploratory field. Currently, there is no universally accepted classification system for their anti-cancer properties. In modern medicine, antibiotics are typically classified based on their mechanism of action, spectrum of antimicrobial activity, or manufacturing process (e.g., natural, synthetic, or semisynthetic) [10]. To provide clarity in this review, we adopt a classification based on their spectrum of antimicrobial activity. This approach is both familiar and widely understood within the scientific community, allowing for a systematic exploration of their anti-cancer potential while maintaining conceptual coherence.

This review aims to analyze the potential of antibiotics as anti-cancer agents, shedding light on their mechanisms of anti-tumor activity across various cancer cell types. Additionally, we will address controversies surrounding the use of antibiotics in cancer treatment and examine novel therapeutic strategies involving original or newly synthesized antibiotics.

## 2. Antibiotics Commonly Utilized in Chemotherapy Protocols

Research on the use of antibiotics in cancer treatment has gained increasing attention, revealing their potential to inhibit tumor growth, induce apoptosis, and prevent metastasis [6]. Several antibiotics traditionally utilized in chemotherapy exhibit diverse mechanisms of action against cancer cells. One prominent group is the anthracyclines, which are known for forming cleavable DNA complexes, inhibiting topoisomerase II activity, and generating ROS. These actions collectively disrupt DNA transcription and replication [11]. Anthracyclines such as idarubicin, daunorubicin, and doxorubicin are widely used in the treatment of leukemia, while epirubicin has demonstrated efficacy against ovarian, breast, and lung cancers [12]. However, anthracyclines, particularly doxorubicin, are notorious for their cardiotoxic effects, which are attributed to their impact on inflammation, oxidative stress, apoptosis, and mitochondrial dysfunction in myocardial cells [13]. Notably, mitoxantrone, an anthracycline analog with reduced cardiotoxicity compared to doxorubicin, has been effectively used against both solid tumors and hematologic malignancies [14,15].

Other antibiotics with established anti-tumor properties include actinomycin and bleomycin [14]. Actinomycin acts through DNA intercalation, preventing RNA synthesis, and is used to treat pediatric cancers such as Wilms’ tumor and rhabdomyosarcoma [16]. Actinomycin use is restricted due to severe adverse effects, including hepatotoxicity, which is rarely associated with hepatic sinusoid occlusion, as well as myelosuppression [17]. Bleomycin, successful against neoplasms like Hodgkin’s lymphoma and testicular cancer, operates by oxidatively cleaving DNA and RNA, damaging mitochondria, and disrupting the G2 phase of the cell cycle [18]. Toxicities attributed to bleomycin include the characteristic drug rash known as flagellate erythema, and the potentially lethal bleomycin-induced pneumonitis [19]. Another key antibiotic, mitomycin, is effective against solid tumors such as anal and bladder carcinomas. Under anaerobic conditions, mitomycin forms covalent DNA linkages and induces alkylation [20], but it also carries a risk of pulmonary toxicity [21].

While the aforementioned antibiotics have proven anti-cancer actions and are utilized in clinical practice, research focusing on different approaches toward cancer treatment is ongoing. A novel conceptual framework for cancer treatment was proposed by Lamb et al., emphasizing the dependence of cancer stem cells (CSCs) on mitochondrial biogenesis. Their work demonstrated that antibiotics like tetracyclines, known to impact mammalian mitochondria as a mild adverse effect, could effectively target CSCs and inhibit various cancers in vitro [22]. This approach highlights the potential for drug repositioning, leveraging the well-established safety profiles and clinical experience of antibiotics in infection management for use in oncology [23].

## 3. Anti-Cancer Potential of Antibiotics Targeting Gram-Positive Bacteria

Several antibiotics developed specifically to address the rise in antibiotic resistance in Gram-positive bacteria have demonstrated anti-cancer qualities in various experimental studies [24]. Vancomycin, a glycopeptide bactericidal antibiotic, has shown promise as an adjunct to radiotherapy in melanoma, lung, and cervical cancer models, both in vivo and in vitro. The combination of radiotherapy and oral vancomycin enhanced antigen presentation in tumors and regional lymph nodes, elevated interferon-gamma (IFN-γ) levels, and increased CD8^+^ T cell activity within the tumor microenvironment (TME). These effects were linked to modifications in the gut microbiota of treated mice, including the elimination of bacteria-producing short-chain fatty acids (SCFAs) [25].

Linezolid, an oxazolidinone-class antibiotic, effectively inhibited the growth of colon cancer cells and induced apoptosis when combined with oxaliplatin. This activity was attributed to mitochondrial dysfunction caused by linezolid, leading to increased ROS in CSCs [26]. Furthermore, linezolid exhibited potential against prostate, breast, and gallbladder cancer cell lines in vitro. Its pro-apoptotic effects were associated with the downregulation of anti-apoptotic genes such as *Bcl-xL* and *Bcl-2* and the inhibition of the Akt signaling pathway. In the same study, teicoplanin and daptomycin, which disrupt bacterial cell wall integrity, demonstrated moderate efficacy against prostate and breast cancer cell lines, respectively [27,28]. Daptomycin has also been reported to selectively inhibit the growth of breast and colon cancer cells in vitro by binding to the human ribosomal protein S19 (RPS19) within the 40S ribosomal subunit. In mice, RPS19 plays a critical role during early developmental stages and is highly expressed in certain colon cancer types [29].

## 4. Anti-Cancer Mechanisms of Antibiotics Targeting Both Gram-Positive and Gram-Negative Bacteria

### 4.1. Beta-Lactams

Beta-lactam antibiotics are one of the earliest and most significant classes of antibacterial agents, characterized by their beta-lactam ring structure. They function by inhibiting bacterial cell wall synthesis, demonstrating activity against both Gram-positive and Gram-negative bacteria [30]. Penicillin G has exhibited notable anti-cancer activity in vitro against cervical cancer and leukemic cell lines by mitigating cell proliferation and inducing cell death. Its mechanisms of action include the downregulation of matrix metalloproteinase (MMP)-11 and signal transducer and activator of transcription 5A (STAT5A). MMPs, initially identified for their role in degrading extracellular connective tissue, are central proteins in cancer progression, influencing cell proliferation, angiogenesis, and apoptosis. Additionally, the upregulation of p53 expression following penicillin G treatment suggests the involvement of an apoptotic pathway. The drug’s cytotoxic effects were dose-dependent, with cancer cells displaying greater sensitivity than non-cancerous cells, underscoring its potential for selective anti-cancer activity [31].

Ceftriaxone, a cephalosporin antibiotic, has been shown to suppress the growth of three lung cancer cell lines by inhibiting Aurora B kinase, a protein that plays a crucial role during mitosis and regulates cell division. These findings have been validated in murine models in vivo, further supporting its anti-cancer potential [32]. Several cephalosporins, including cefuroxime, cefotaxime, cefmetazole, and ceftazidime, selectively induced ferroptosis in nasopharyngeal carcinoma cells both in vitro and in vivo by upregulating *HMOX1*, a gene involved in oxidative stress response. Additional proposed anti-cancer mechanisms of cephalosporins include apoptosis induction, angiogenesis and metastasis inhibition, and cell cycle arrest. These effects were supported by the favorable regulation of genes such as *DDIT3*, *GADD45A*, and *JUN*, along with the downregulation of *MUC1* and *FZD10* [33]. Metal complexes of cefotaxime demonstrated strong, dose-dependent cytotoxicity against liver cancer cells. Researchers attributed this effect to the antioxidant properties of the compounds, suggesting a potential dual role in cytotoxicity and hepatoprotection [34].

### 4.2. Sulfonamides

Sulfonamides are bacteriostatic antibiotics that inhibit bacterial DNA replication and growth by interfering with folic acid synthesis [35]. These compounds have also demonstrated potential as anti-cancer agents in both in vitro and in vivo models. Sulfamethoxazole induced necrosis in four distinct cancer cell lines—colon, breast, prostate, and hepatocellular—in vitro. Its anti-tumor effect was significantly enhanced when combined with quercetin, a dietary polyphenol found in fruits and vegetables, with apoptosis being the primary mechanism of action. The swift towards the apoptotic pathway was attributed to the addition of quercetin and was confirmed by elevated caspase-3 levels both in vivo and in vitro. The suggested mechanism involves the antioxidant and protective properties of the combination, evidenced by increased levels of antioxidant enzymes, including superoxide dismutase (SOD), glutathione reductase (GSH) activity, total antioxidant capacity (TAC), and catalase (CAT). These changes were correlated with a reduction in malondialdehyde (MDA), indicating decreased lipid peroxidation in treated cells. Suppressed ROS levels subsequently led to reduced nuclear factor kappa-light-chain-enhancer of activated B cells (NF-κB) expression, a key inhibitor of apoptosis. Furthermore, the sulfamethoxazole-quercetin combination induced cell cycle arrest at the G2/M phase and potentially inhibited metastatic progression [36].

The combination of sulfamethoxazole with selenium showed efficacy in preventing liver injury in mice injected with diethyl-nitrosamine (DENA), a potent carcinogen that induces hepatocarcinogenesis. The treatment reversed liver injury, as evidenced by reduced serum liver enzyme levels, along with decreased total cholesterol and triglycerides—markers typically elevated in rapidly proliferating cancer cells. Histological analyses revealed that mice treated with this combination lacked the hepatic disorganization observed in untreated controls [37]. Moreover, trimethoprim-sulfamethoxazole (TMP-SMX) demonstrated efficacy in treating melanoma in immunodeficient mice and showed potential as an immune-enhancing agent. Apart from promoting apoptosis through the activation of caspases-3, -8, and -9, TMP-SMX increased tissue infiltration by mast cells and boosted the release of allergy-associated mediators. It also elevated levels of pro-inflammatory cytokines such as tumor necrosis factor-alpha (TNF-α), interleukin (IL)-2, and IL-6 [38].

### 4.3. Aminoglycosides

Aminoglycosides are broad-spectrum antibiotics that inhibit bacterial protein synthesis. Despite their significant side effects, including nephrotoxicity and ototoxicity, they remain critical tools in combating multidrug-resistant (MDR) bacteria [39]. Emerging research has also revealed notable anti-cancer qualities associated with this class of antibiotics.

Neomycin has been shown to inhibit glioma cell proliferation in vitro by downregulating cyclin D1, a gene regulated by NF-κB that is essential for the G1-to-S phase transition in the cell cycle. Additional mechanisms of neomycin-mediated cell cycle arrest include the inhibition of p42/44 mitogen-activated protein kinase (MAPK) and cAMP response element-binding protein (CREB)-directed transcription pathways. The MAPK system plays a crucial role in growth factor-mediated signal transduction in both normal and glioma cells, while CREB is a vital transcription factor involved in cell survival regulation [40,41]. Furthermore, neomycin has demonstrated anti-angiogenic effects, initially observed in human umbilical vein endothelial (HUVE) cells, by inhibiting the nuclear translocation of angiogenin, a key inducer of neovascularization, and reducing the amount of Von Willebrand factor in the cellular matrix [42]. These anti-angiogenic properties are further supported by studies on neamine, a neomycin derivative, which suppressed growth in two oral cancer cell lines in vivo through similar inhibition of angiogenin nuclear translocation [43].

Gentamicin has also demonstrated diverse anti-cancer mechanisms. By upregulating the expression and activity of acid sphingomyelinase (aSMase), gentamicin altered sphingomyelin metabolism, resulting in increased apoptosis and reduced proliferation of gastric cancer cells in vitro [44]. Acid sphingomyelinase, responsible for cleaving sphingomyelin, a sphingolipid involved in signal transduction and cell differentiation, has been implicated in both cancer development and apoptosis regulation. Gentamicin’s pro-apoptotic effects have also been observed in non-Hodgkin’s T cell human lymphoblastic lymphoma and hepatoma cell lines. Additionally, gentamicin-induced production of ROS has generated interest in its potential use against non-small-cell lung cancer (NSCLC), particularly in combination with traditional chemotherapy agents like vinblastine [44,45,46]. Clinical evidence supports gentamicin’s utility in cancer treatment. In a clinical trial involving rectal cancer patients, locally administered gentamicin post-surgery not only reduced early post-operative complications but also decreased distant metastases and improved overall survival (OS) [47]. Further research by Frumkin et al. suggests that gentamicin binds to human ribosomes and promotes translational read-through of premature stop codons, thereby restoring truncated, non-functional tumor suppressor proteins in cancer cells [48,49].

Puromycin, a natural aminoglycoside, with limited clinical use has also shown promise in cancer therapy. It was found to enhance the efficacy of gemcitabine against three resistant cancer cell lines, including lung and liver cancer cell lines. This synergistic effect is attributed to the downregulation of key signaling pathways, including Akt, mTOR, MAPK, and extracellular signal-regulated kinase (ERK) 1. The discovery of puromycin as a potential anti-cancer agent was facilitated by high-throughput screening (HTS), an advanced technique with significant potential in cancer treatment development [50].

## 5. Anti-Cancer Properties of Antibiotics Active Against Atypical Bacteria

### 5.1. Macrolides

#### 5.1.1. Traditional Macrolides

Macrolides represent a diverse class of antibiotics that primarily function by inhibiting bacterial protein synthesis through binding to the 50S ribosomal subunit [51]. Beyond their antimicrobial efficacy, macrolides have garnered significant attention for their anti-cancer potential. Evidence suggests that these compounds can modulate key cellular processes in cancer cells, including apoptosis, autophagy, and cell cycle regulation, positioning them as promising candidates for drug repurposing.

Erythromycin, one of the earliest macrolides, has demonstrated anti-tumor activity in human neuroblastoma cells by initiating apoptosis. This effect is mediated by upregulating the cyclin-dependent kinase inhibitor p21(^WAF1/Cip1^), which induces S-phase cell cycle arrest, and downregulating the cancer-associated *c-Myc* gene, leading to intracellular calcium overload. Additionally, erythromycin enhances IL-4 production, which augments macrophage-mediated tumoricidal activity, and inhibits the human Ether-à-go-go-related gene (hERG) potassium channel, overexpressed in several cancer types. These mechanisms have been validated in vivo using Ehrlich ascites carcinoma (EAC) and P388 leukemia cells and in vitro in colon, breast, and lung cancer models [52,53,54].

Clarithromycin has shown similar promise, particularly in CRC models. It modifies autophagy, a survival mechanism often exploited by cancer cells, by initially stimulating autophagy but subsequently causing autophagy exhaustion. This process, driven by clarithromycin’s binding to the hERG1 potassium channel and inhibition of PI3K interaction, culminates in apoptotic cell death. In combination with 5-fluorouracil (5-FU), clarithromycin enhances chemotherapy efficacy, highlighting its potential as part of combinatory regimens [55].

Azithromycin, a second-generation macrolide, exhibits broad-spectrum anti-cancer activity against diverse malignancies, including chronic myeloid leukemia (CML), glioblastoma (GB), hepatocellular carcinoma (HCC), and lung cancer. Proposed mechanisms include caspase-3 and -7 activation in leukemia cells, downregulation of MAPK3 and rapidly accelerated fibrosarcoma 1 (RAF1) mRNA in liver cancer, and upregulation of autophagy markers microtubule-associated protein 1A/1B-light chain 3B type II (LC3B-II) and p62 in lung and colon cancer cells. These effects have been observed in monotherapy and in combination with agents such as doxorubicin, imatinib, and sorafenib, with promising results [56,57,58,59,60,61]. Additional studies illuminate azithromycin’s anti-inflammatory and anti-angiogenic properties, as seen in increased TNF receptor type 1 expression in liver cancer cells and reduced vascular endothelial growth factor (VEGF) and VEGF receptor 2 expression in lung cancer models [61,62,63]. Remarkably, azithromycin may also target glioma stem cells by inhibiting mitochondrial biogenesis and oxidative phosphorylation (OXPHOS), thereby thwarting cancer cell quiescence and enhancing susceptibility to cytotoxic drugs [56,64].

#### 5.1.2. Novel Macrolides

Rapamycin, also known as sirolimus, is a macrolide antibiotic that is commonly administered for prophylaxis of renal graft rejection [65]. Beyond its immunosuppressive properties, rapamycin and its analogs, such as everolimus and temsirolimus, have been approved for the treatment of solid tumors, reflecting their significant potential in oncology [66]. Numerous studies have demonstrated rapamycin’s anti-cancer effects in vivo, where it has been shown to prevent or delay the onset of various cancers, including lung cancer and head and neck squamous cell carcinoma, in mice exposed to carcinogens. This anti-cancer activity is attributed to its inhibition of the mTOR pathway, which is frequently dysregulated in cancer [66,67,68].

Furthermore, rapamycin treatment in genetically modified, cancer-prone mice has consistently resulted in extended lifespan, suppression of carcinogenesis, and delayed onset of aging-related phenotypes. The types of cancers prevented through rapamycin administration include prostate, intestinal, ovarian, and neuroendocrine tumors, as well as head and neck squamous cell carcinomas [69,70,71]. Intriguingly, similar findings have been observed in wild-type mice, where rapamycin administration led to increased overall life expectancy, suggesting its ability to slow aging processes and mitigate the risk of age-related cancers. Clinical evidence also supports rapamycin’s protective role, with prospective studies in organ transplant recipients indicating a lower risk of overall cancer occurrence among those receiving the drug. Notably, rapamycin is increasingly being used off-label by individuals worldwide as part of experimental approaches to slow aging, reflecting a growing interest in its dual benefits for cancer prevention and longevity enhancement [66].

Lastly, rokitamycin has exhibited in vitro anti-cancer effects against T cell leukemia and myelogenous leukemia by inducing apoptosis through mitochondrial transmembrane potential reduction, cytochrome c release, and caspase-9/-3 activation. This p53-independent mechanism makes rokitamycin a potential therapeutic option for malignancies harboring p53 mutations, a common feature in treatment-resistant malignancies [72].

### 5.2. Tetracyclines

Tetracyclines are a broad class of antibiotics that function by binding to the 30S ribosomal subunit, thereby interrupting protein synthesis and inhibiting bacterial cell growth [73]. Among antibiotic classes, tetracyclines stand out for their extensively studied potential in cancer treatment, with numerous studies elucidating their diverse mechanisms of anti-tumor qualities.

#### 5.2.1. Second Generation Tetracyclines

Doxycycline has shown significant promise against various tumor types. Its proposed mechanisms of action include the prevention of metastasis and elimination of CSCs, as evidenced by the in vitro downregulation of CSC-associated biomarkers such as octamer-binding transcription factor 4 (OCT-4), aldehyde dehydrogenase 1 (ALDH1), B cell-specific Moloney murine leukemia virus integration site 1 (Bmi-1), homeobox protein NANOG (hNanog), and neurogenic locus notch homolog protein (Notch) in breast CSCs. Doxycycline’s selective action against CSCs is attributed to the combination of CSC overexpression of mitochondrial proteins and the drug’s inhibition of mitochondrial function [74,75]. Studies further demonstrate that doxycycline disrupts mitochondrial function and OXPHOS, as shown by its ability to increase sensitivity to hypoxia and glucose uptake in glioma cell lines [76].

Additionally, doxycycline-mediated activation of the endothelial Wnt/β-catenin pathway has been implicated in the suppression of glioma cell growth and vascular proliferation, as well as the inhibition of metastatic spread in gastric cancer models. This process involves upregulation of interferon regulatory factor-1 (IRF-1), linking embryonic developmental pathways to cancer progression [77,78,79]. In prostate cancer cells, doxycycline induced apoptosis when combined with doxorubicin by increasing B-cell lymphoma 2 (Bcl-2) associated X protein (BAX) protein expression and reducing Bcl-2 expression, key regulators of p53-mediated apoptosis. Furthermore, doxycycline inhibited angiogenesis in pancreatic cancer cells and blocked epithelial-to-mesenchymal transition (EMT) in HCC models by downregulating MMP-2 and MMP-9 [74,80,81].

Doxycycline also enhances immune system anti-tumor activity. It prevents the removal and increases the expression of tumor cell ligands recognizable by natural killer (NK) cells and cytokine-induced killer (CIK) cells in ovarian, breast, and CRC cells [82]. In breast cancer cells, doxycycline interacts with integrin ανβ3, leading to inhibition of ERK1/2 phosphorylation, a signaling event that halts further cell development [83,84]. Concurrently, doxycycline suppresses programmed death-ligand 1 (PD-L1) expression, thereby enhancing immune surveillance and reducing cancer cell immune evasion [84].

Notable effects of doxycycline have been observed in osteosarcoma, where it activates Notch1, a transmembrane receptor and cell differentiation regulator, and inhibits the PI3K/Akt/mTOR signaling pathway, resulting in S-phase arrest, apoptosis, and autophagy of cancer cells [85]. Similarly, in melanoma models, doxycycline induces apoptosis and upregulates markers of autophagy, both effects associated with increased ERK1/2 expression, a mechanism different to the aforementioned ERK1/2 inhibition in breast cancer cells [84]. The ERK1/2 pathway appears to play a significant role in cell proliferation and differentiation, and its inhibition has been linked to prevention of tumor growth and metastasis [86]. Reports of anthracycline-induced ERK1/2 upregulation associated with resistance development in breast cancer cells highlight the current knowledge gap about the finer points of this pathway and the need for further research [87]. Additionally, doxycycline enhances the expression of microphthalmia-associated transcription factor (MITF), a transcription factor involved in melanin synthesis and potentially linked to cell cycle regulation in melanoma [88].

Doxycycline’s anti-inflammatory properties contribute to its anti-tumor effects. In lung and prostate cancer models, doxycycline inhibits the formation of the NOD-like receptor P3 (NLRP3) inflammasome, reducing inflammation and arresting tumor growth [89]. In murine breast cancer models, treatment with doxycycline was associated with reduced plasma levels of pro-inflammatory cytokines such as IL-1β, IL-6, IL-9, VEGF, and granulocyte colony-stimulating factor (G-CSF) [90]. Combining doxycycline with other anti-cancer agents has yielded synergistic benefits. For instance, it has been paired with oncolytic vaccinia viruses targeting breast cancer cells, immune checkpoint inhibitors, and anti-angiogenic drugs. Notably, doxycycline reduces skin toxicities associated with immune checkpoint inhibitors (ICIs) and anti-epidermal growth factor (EGF) receptor therapies, improving tolerability for cancer patients [75,91,92].

Minocycline, though less extensively studied, has demonstrated potential anti-tumor effects. It inhibits the NF-κB signaling pathway in breast and ovarian cancer cells, thereby reducing gene expression associated with cancer progression [93]. However, minocycline appears less effective than doxycycline against melanoma cells [88]. Like doxycycline, minocycline has been shown to mitigate skin toxicities related to anti-EGF receptor therapies and improve systemic symptoms such as anorexia and pain in head and neck cancer patients [94,95].

#### 5.2.2. Third Generation Tetracyclines

Tigecycline has emerged as a potent agent against several cancers. It induces G0/G1 phase arrest in pancreatic ductal adenocarcinoma (PDAC) cells by downregulating cell cycle-associated proteins, including CDK2 and CDK4, and inhibits EMT processes [96]. In neuroblastoma cells, G1 arrest was linked to the inhibition of the Akt pathway [97]. Tigecycline also promotes autophagy in gastric cancer cells by increasing AMP-activated protein kinase (AMPK) phosphorylation and decreasing mTOR phosphorylation [98]. Additionally, it has shown efficacy against retinoblastoma, HCC, and kidney cancer, primarily through the inhibition of mitochondrial respiratory complexes I and IV, leading to suppressed mitochondrial translation [99]. Further mechanisms of tigecycline include inhibition of the Wnt/β-catenin pathway in CRC and cervical squamous carcinoma models, as well as suppression of the STAT3 signaling pathway, which regulates CRC proliferation and metastasis. Notably, tigecycline also modulates gut microbiota, reducing pro-inflammatory cytokines such as TNF-α and IL-16, which are associated with cancer progression [99,100].

**Figure 1:**
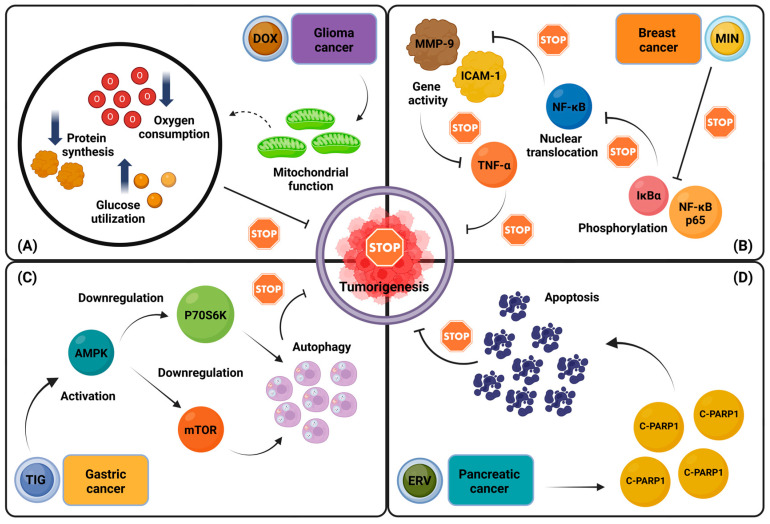
Differential anti-cancer mechanisms of tetracyclines across experimental cancer models. This figure depicts the anti-cancer effects of four tetracycline derivatives—doxycycline, minocycline, tigecycline, and eravacycline—on glioma, breast, gastric, and pancreatic cancer models, respectively. (**A**). Doxycycline inhibits mitochondrial protein synthesis in glioma cells, resulting in metabolic reprogramming characterized by reduced oxygen consumption and increased glucose utilization. (**B**). Minocycline disrupts TNF-α-induced cell fusion in breast cancer cells by inhibiting NF-κB activation, preventing IκBα and NF-κB-p65 phosphorylation, nuclear translocation of NF-κB, and subsequent expression of MMP9 and ICAM1. (**C**). In gastric cancer cells, tigecycline promotes autophagy-mediated growth inhibition by activating the AMPK pathway, leading to the suppression of downstream targets such as mTOR and p70S6K. (**D**). Eravacycline treatment in pancreatic cancer cells induced dose-dependent apoptosis, as demonstrated by the significant upregulation of C-PARP1 [76,93,98,101]. Abbreviations: AMPK: AMP-activated protein kinase; C-PARP1: Cleaved poly (ADP-ribose) polymerase 1; DOX: Doxycycline; ERV: Eravacycline; ICAM-1: Intercellular adhesion molecule-1; IκBα: Inhibitor of nuclear factor kappa-B kinase alpha; MIN: Minocycline; MMP-9: Matrix metalloproteinase-9; mTOR: Mechanistic target of rapamycin; NF-κΒ: Nuclear factor kappa-light-chain-enhancer of activated B cells; p70S6K: p70 ribosomal protein S6 kinase; TIG: Tigecycline; TNF-α: Tumor necrosis factor-alpha. ↑: Increase; ↓: Decrease. Created in BioRender. Kounatidis, D. (2024), https://BioRender.com/c76h343 (assessed on 24 December 2024).

When combined with gemcitabine, tigecycline enhances apoptotic activity and improves efficacy against PDAC cells in vitro and in vivo [96]. Other successful combinations include pairing tigecycline with tyrosine kinase inhibitors (TKIs) for CML and cisplatin for HCC and chemoresistant ovarian cancer [73,76]. Eravacycline, a synthetic tetracycline related to tigecycline, has also shown promise in vitro against PDAC cells, inducing apoptosis at levels comparable to cisplatin and gemcitabine [101]. Figure 1 illustrates the anti-cancer effects of four distinct tetracyclines across four experimental models, each targeting a different type of cancer.

### 5.3. Quinolones

Quinolone antibiotics exert their antibacterial effects by inhibiting DNA gyrase and type IV topoisomerase, thereby disrupting bacterial DNA synthesis. Among the four quinolone generations, second- and third-generation compounds have been extensively studied for their potential as anti-cancer agents due to their inhibitory effects on mammalian DNA topoisomerase I, topoisomerase II, and DNA polymerase [102].

Ciprofloxacin and ofloxacin have demonstrated efficacy in eliminating three bladder transitional carcinoma cell lines at concentrations achievable in urine following oral administration. Notably, they exhibited enhanced activity against p53-positive cells, which are typically resistant to chemotherapy, and increased the toxicity of doxorubicin. These quinolones act similarly to anthracyclines, effectively killing cancer cells exfoliated during transurethral procedures while preventing their reimplantation and subsequent tumor recurrence [102]. Efforts to improve ciprofloxacin’s cellular uptake and reduce its effective concentration have included its combination with other molecules. For example, ciprofloxacin, combined with quaternary ammonium-functionalized polycarbonate, was lethal to breast and HCC cells by fostering a positive feedback loop for enhanced polymer and drug internalization [103]. Similarly, pairing ciprofloxacin with saturated and unsaturated fatty acids (FAs) proved effective against primary and metastatic colon cancer as well as metastatic prostate cancer cells. This combination reduced IL-6 secretion from cancer cells and induced late apoptosis [104].

Among newer fluoroquinolones, moxifloxacin has shown significant anti-cancer activity both in vitro and in vivo. It enhances the action of etoposide, a topoisomerase II inhibitor, on colon carcinoma cells by promoting DNA-topoisomerase II adduct formation and enhancing apoptosis. Additional mechanisms of moxifloxacin include inhibiting VEGF secretion and suppressing etoposide-induced pro-inflammatory IL-8 secretion [105]. These dual effects, amplifying etoposide’s cytotoxicity while mitigating its pro-inflammatory effects, have been observed in acute monocytic and T cell leukemia models [106]. Moxifloxacin has also been proven effective in colon carcinoma xenografts in Severe Combined Immunodeficiency (SCID) mice when combined with irinotecan, enabling lower irinotecan doses. This study confirmed both moxifloxacin’s anti-topoisomerase II activity and its anti-angiogenic effects in vivo [107]. Significantly, moxifloxacin is among the few antibiotics with demonstrated anti-cancer efficacy in a phase II clinical trial. In a study involving 30 patients with metastatic breast cancer and stable disease but increasing tumor size, a weekly course of moxifloxacin yielded a median progression-free survival of 6.6 months, with overall and clinical response rates of 23.3% and 46.7%, respectively [108]. Moxifloxacin was particularly effective in HR-negative (hormone receptor-negative) and HER2-positive (human epidermal growth factor receptor 2-positive) patients, potentially due to its ability to eradicate tumor-associated microbiota that contributed to tumor progression, chemoresistance, and angiogenesis [108].

Gatifloxacin, a third-generation quinolone, successfully inhibited the growth of two pancreatic cancer cell lines in vitro. Its proposed mechanisms of action include downregulation of the Akt pathway, increased sensitivity to conventional chemotherapy agents like gemcitabine, and cell cycle arrest at the S and G2 phases without inducing apoptosis. Molecular pathways implicated in S-phase arrest include transforming growth factor-beta 1 (TGF-β1)-mediated p21 activation and direct activation of p27, whereas G2-phase arrest was found to be p53-dependent [109].

**Figure 2:**
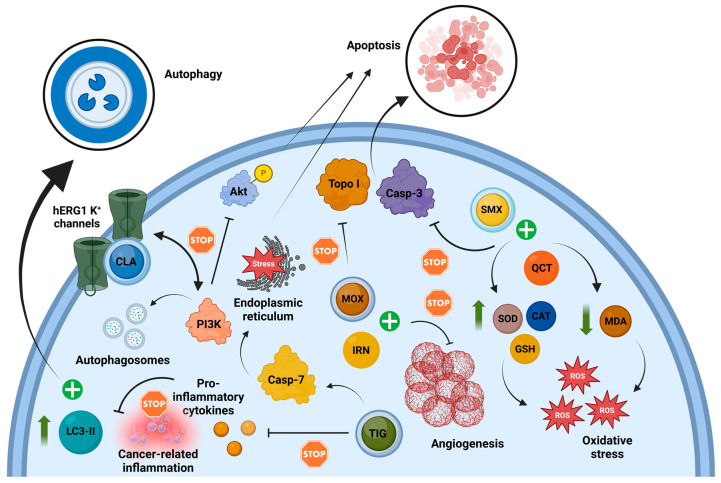
The anti-cancer effects of different commonly prescribed antibiotics in combating colon cancer. Sulfamethoxazole combined with quercetin enhances antioxidant defenses, as evidenced by increased levels of SOD, GSH, and CAT, while reducing MDA levels. This combination also promotes apoptosis through upregulation of caspase-3. Moxifloxacin facilitates apoptosis by inhibiting topoisomerase I and, when combined with irinotecan, alleviates angiogenesis. Tigecycline induces apoptosis via caspase-7 upregulation, ER stress induction, and downregulation of pro-inflammatory cytokines, debilitating cancer-associated inflammation. Clarithromycin disrupts autophagic flux, causing autophagy exhaustion and apoptosis by binding to the closed conformation of hERG1 potassium channels. This interaction prevents binding to the p85 subunit of PI3K, reducing Akt phosphorylation and activating apoptotic pathways. Disrupted autophagy is evidenced by autophagosome accumulation and modulation of LC3-II [36,55,99,107]. Abbreviations: Akt: Protein kinase B; Casp: Caspase; CAT: Catalase; CLA: Clarithromycin; GSH: Glutathione; hERG1: Human ether-à-go-go-related gene 1; IRN: Irinotecan; LC3-II: Microtubule-associated protein 1A/1B-light chain 3 type II; MDA: Malondialdehyde; MOX: Moxifloxacin; PI3K: Phosphoinositide 3-kinase; QCT: Quercetin; SMX: Sulfamethoxazole; SOD: Superoxide dismutase; TIG: Tigecycline. Topo I: Topoisomerase. ↑: Increase; ↓: Decrease. Created in BioRender. Kounatidis, D. (2024), https://BioRender.com/v81b235 (assessed on 24 December 2024).

The data presented above demonstrate that commonly used antibiotic drugs can exhibit significant anti-cancer properties by targeting various mechanisms across different types of cancers. Figure 2 provides a visual representation of the potential intervention points of these antibiotic drugs within a hypothetical experimental model of colon cancer, summarizing the findings discussed in the preceding sections.

## 6. Anti-Cancer Effects of Antibiotics Targeting Anaerobic Bacteria

Metronidazole, a widely used antibiotic effective against anaerobic bacteria and protozoa, has shown promising results in oncology by complementing established treatments. In vivo, metronidazole enhanced the efficacy of radiotherapy by targeting hypoxic anaplastic cells, likely through the formation of free radicals that mimic its antibacterial mechanism of action [110,111]. More recently, metronidazole demonstrated effectiveness against gemcitabine-resistant cholangiocarcinoma cells in vitro, where it enhanced chemosensitivity and inhibited cell migration. These effects were linked to the suppression of aldehyde dehydrogenase (ALDH) activity, a well-recognized CSC marker, suggesting a disulfiram-like mechanism [112]. Additionally, silver complexes of metronidazole have exhibited potent cytotoxicity against HCC and CRC cell lines in vitro. The combination of metronidazole and silver synergistically induced DNA damage and ROS production, significantly outperforming the effects of either compound alone [113].

The stability of these complexes (demonstrated here with metronidazole) generally surpasses that of silver salts such as AgSO_3_ and AgNO_3_. However, the researchers have not provided an exact calculation of the dissociation constant (Kₑ) [113]. Despite showing promise in both in vitro and in vivo studies due to their antimicrobial, anti-cancer, and anti-inflammatory properties, silver complexes have not yet gained widespread clinical approval for most human indications. While silver ions are inherently toxic, nanoscale silver exhibits broad-spectrum antimicrobial and anti-cancer activity with fewer toxicological side effects compared to ionic silver. Nevertheless, the lack of selectivity in silver complexes remains a significant challenge, and their associated toxicity risks must be carefully considered, as these factors limit their clinical applicability [114].

## 7. Anti-Tumor Potential of Rifampicin

The semi-synthetic antibiotic rifampicin is a cornerstone of tuberculosis treatment. Oral rifampicin successfully inhibited growth and spread of primary lung and colon cancer in murine models via suppression of angiogenesis. Rifampicin-induced downregulation of angiogenesis-associated genes and eradication of microvascular endothelial cells was superior to endostatin. Notably, after a mean follow-up period of 97.3 months, only a single patient out of 6 patients with hepatitis C-induced cirrhosis resistant to interferon-alpha (IFN-α) treated a 150–300 mg daily dose of rifampicin, developed HCC [115,116]. The potential of combining rifampicin with vinblastine to treat resistant leukemia and liver cancer cells in vivo was examined in an earlier study. The addition of rifampicin augmented the accumulation and activity of vinblastine by diminishing its export out of cells [117].

Based on the above, it is evident that experimental pharmacology has significantly highlighted the potential anti-cancer activity of antibiotics commonly utilized in clinical practice, offering promising prospects for the potential application of these findings in clinical settings. In Table 1, we provide examples of antibiotics from each major category, highlighting their anti-cancer potential. The table outlines their mechanisms of action, along with their effects on various cancer cell lines, and key cellular processes such as apoptosis, cytokine release, and cytotoxicity.

## 8. Anti-Cancer Mechanisms of Miscellaneous Uncommon Antibiotics

Various antibiotics outside the traditionally studied classes have shown promising anti-cancer potential through diverse mechanisms. Ascofuranone, an antibiotic initially developed to target *Trypanosoma brucei*, the causative agent of sleeping sickness, demonstrated anti-tumor activity in mice challenged with L-1210 leukemia cells. The anti-cancer effect was mediated through the regulation of lipid metabolism; however, the timing of drug administration was crucial for its efficacy [118]. Monensin, a polyether antibiotic primarily used in veterinary medicine, exhibited anti-cancer properties when combined with erlotinib for the treatment of triple-negative breast cancer (TNBC) cells. The combination suppressed the growth of breast CSCs by directly inhibiting both the EGFR/ERK and PI3K/Akt signaling pathways in CSCs, both in vitro and in vivo. This led to reduced CSC proliferation and promoted apoptosis, as evidenced by a decrease in specific CSC markers such as SOX2 and CD133 [119].

Cordycepin, a nucleoside antibiotic isolated from *Cordyceps militaris*, exerts its anti-cancer effects through multiple mechanisms. In breast cancer cells, cordycepin induces apoptosis via increased BAX expression. In leukemia cells, it upregulates p53, while in oral cancer cells, it activates the c-Jun N-terminal kinase (JNK) and caspase pathways [120,121,122]. In leukemia, cordycepin also causes S-phase cell cycle arrest by suppressing the expression of cyclin A2, cyclin E, and cyclin-dependent kinase 2 (CDK2). Additionally, it increases ROS production in bladder cancer cells and inhibits both angiogenesis and metastasis in CRC cells through regulation of prostaglandin E2 receptor 4 (EP4) expression and the AMPK-CREB signaling pathway [120,123,124]. Cordycepin has also been shown to prevent the metastasis and invasion of liver cancer cells by reducing C-X-C chemokine receptor 4 (CXCR4) expression, and it enhances macrophage-mediated phagocytosis of CRC cells by blocking the immune checkpoint CD47 [125,126]. Lastly, mupirocin, typically used as a topical antibiotic, demonstrated anti-cancer potential when encapsulated in PEGylated nanoliposomes (nano-mupirocin). This formulation effectively targeted and eradicated tumor-associated *Fusobacterium nucleatum* bacteria in breast cancer models, suggesting its potential in microbiota-related oncogenesis [127].

## 9. Challenges and Controversies in Antibiotic Use for Cancer Treatment

Antibiotics hold significant promise in cancer treatment, yet their application is marred by several challenges and controversies. Benefits must be weighed against risks such as disruption of gut microbiota, microbial resistance, and interference with other cancer therapies, which have led to their characterization as a “double-edged sword” [4]. Additionally, antibiotics are associated with other adverse effects, including the emergence of antibiotic-resistant microbial species and damage to both male and female reproductive systems. Although these effects are not directly related to their anti-cancer potential, they impose significant limitations on their therapeutic applications [6,128].

Regardless of whether antibiotics are used to target bacteria or cancer, mitigating their toxicity is essential to preserve their efficacy. This can be achieved through various therapeutic strategies, such as the combined use of synergistic antibiotics or biocides, as well as the incorporation of plant-derived substances, essential oils, and small molecules—both naturally occurring and synthetic—that act directly against pathogens. Novel approaches are enhancing the antibacterial potential of antibiotics. These include bacterial genome modification and the removal of microbial resistance genes using clustered regularly interspersed short palindromic repeats-associated protein (CRISPR-Cas), as well as drugs targeting exotoxins. A notable example is bezlotoxumab, a monoclonal antibody targeting toxin B of *Clostridioides difficile*. However, the effectiveness of these strategies in cancer treatment remains unclear [129,130].

### 9.1. Carcinogenic Potential of Specific Antibiotics

Some antibiotics exhibit carcinogenic tendencies. For instance, chloramphenicol, an older antibiotic that inhibits protein synthesis by binding to the 70S ribosome, has been repeatedly linked to carcinogenesis and cancer progression. In in vivo murine models, chloramphenicol induced apoptosis and abnormal differentiation of T cells into lymphoblastic leukemia-like cells. Additionally, in vitro studies demonstrated its ability to increase MMP-13 expression, facilitating MMP-13-associated cancer cell invasion. The underlying mechanism appears to involve mitochondrial stress and subsequent mitochondria-to-nucleus stress signaling via JNK and PI3K upregulation. Other studies have associated chloramphenicol-induced mitochondrial stress with p21 activation and the prevention of apoptosis. These findings raise potential concerns about other classes of antibiotics that similarly affect mitochondrial function [131,132,133].

Contrary to the findings discussed in a previous section, doxycycline has been associated with chronic intestinal inflammation and the promotion of carcinogenesis and metastasis in CRC rat models. This pro-inflammatory effect is mediated by the activation of NF-κB and the downregulation of p53 and caspase pathways. While doxycycline-induced gastrointestinal injury has been documented in humans, its link to cancer remains limited to experimental data [134,135].

### 9.2. Antibiotics, Gut Microbiota Disruption, and Cancer Risk

The gut microbiota plays a pivotal role in maintaining physiological homeostasis, including nutrient metabolism and immune system regulation. Disruption of the gut microbiota, termed dysbiosis, has been implicated in a range of diseases, including inflammatory and neoplastic conditions. Dysbiosis can facilitate the proliferation of pro-carcinogenic bacteria, such as *Fusobacterium* spp., which are associated with CRC. It also diminishes the immune-stimulatory benefits of commensal gut bacteria, contributing to systemic inflammatory responses and increasing the risk of conditions like inflammatory bowel disease (IBD) and cancer [136,137].

Antibiotic use is a significant trigger of gut dysbiosis, and evidence links it to cancer development [138]. A nested case–control study from the United Kingdom demonstrated a slight but notable increase in CRC risk with penicillin exposure over a decade prior, particularly after repeated courses. Additional studies have corroborated these findings in different contexts. For instance, diabetic patients treated with anti-anaerobic antibiotics exhibited an elevated CRC risk, while data from Finnish cancer registries indicated a correlation between higher antibiotic use and increased incidences of solid tumors such as breast and lung cancers. Nevertheless, the retrospective nature of these studies introduces inherent limitations [139,140,141]. Furthermore, a meta-analysis of 25 observational studies involving approximately 8 million patients identified an 18% higher overall cancer risk linked to antibiotic use. This association was particularly pronounced in hematological, lung, pancreatic, and genitourinary cancers and was further amplified with prolonged antibiotic exposure [142]. Conversely, prior antibiotic use has been weakly associated with lung cancer development in prospective studies, though confounding factors such as smoking and chronic obstructive pulmonary disease complicate interpretations [143].

Antibiotics also appear to impair cancer treatment efficacy, particularly in the context of ICIs. A meta-analysis of 2740 cancer patients treated with ICIs found that antibiotic use before or during therapy was associated with reduced overall survival (OS) and progression-free survival (PFS) [144]. Since gut microbiota modulates the tumor microenvironment and enhances anti-tumor immune responses, antibiotic-induced dysbiosis may undermine these therapeutic effects [145]. Experimental studies further elucidate this interaction. Kuczma et al. demonstrated that antibiotic prophylaxis reduced T cell-mediated responses against B-lymphoma cells in mice treated with cyclophosphamide, a known immune enhancer. Additionally, the therapeutic efficacy of tumor-specific CD4^+^ T cells was compromised in antibiotic-exposed mice with CRC [146]. Similarly, Routy et al. linked microbiota richness to improved responses to programmed cell death protein 1/programmed cell death ligand 1 (PD-1/PD-L1) monoclonal antibodies in patients with NSCL and renal cell carcinoma. They identified *Akkermansia muciniphila* as a key commensal species supporting immunotherapy efficacy, with oral supplementation reversing resistance to PD-1 blockade in mice [147]

## 10. The Emerging Roles of Antibiotic Derivatives in Cancer Therapy: Perspectives for Future Clinical Applications

The repurposing and modification of antibiotics hold considerable promise in advancing cancer treatment. Research highlights the potential of both traditional antibiotics and synthetic molecules derived from them to combat cancer through innovative mechanisms, reduced toxicity, and synergistic effects when combined with existing therapies.

### 10.1. β-Lactam-Based Innovations

Modified β-lactams, a hallmark of antibiotic innovation, demonstrate diverse anti-cancer mechanisms. Cephalosporins, when utilized as prodrugs in combination with chemotherapeutics like nitrogen mustards and doxorubicin, exhibit comparable efficacy to standalone treatments while reducing toxicity in vitro [148,149]. A notable modification involves integrating a β-lactam ring scaffold into combretastatin A-4 (CA-4), a microtubule-targeting agent (MTA). This innovation prevents glucuronidation, overcoming resistance in breast and colon cancer cells [150]. Similarly, β-lactam hybrids, including N-thiolated-β-lactams and α-quaternary chiral lactam derivatives, target cancer cells via diverse mechanisms. These include poly (ADP-Ribose) polymerase (PARP) inhibition, AMPK activation in colon cancer, apoptosis induction in advanced breast cancer, and inhibition of prostate-specific antigen (PSA) and heat shock protein 90 (Hsp90) [151,152,153,154,155,156,157,158]. Additionally, phenethylamine-based β-lactam derivatives have demonstrated dual efficacy against CRC cell lines and concurrent infections, potentially addressing cancer and infection synergistically [159].

### 10.2. Non-β-Lactam Antibiotic Derivatives

Beyond β-lactams, quinolone and quinoline derivatives selectively induce apoptosis in cervical, bone marrow, and breast cancer cells, with breast cancer cells showing heightened sensitivity [160]. Tunicamycin, a glycosylation inhibitor, effectively targets MDR gastric cancer cells through Endoplasmic reticulum (ER) stress induction and autophagy in vitro [161]. Similarly, epothilones, a class of macrolide compounds targeting microtubules, arrest the cell cycle in multiple myeloma resistant to paclitaxel, while chemically modified tetracycline COL-3 enhances paclitaxel efficacy against breast cancer and mitigates neuropathic pain in vivo [162,163]. Moreover, synthetic sulfamethoxazole derivatives show selective inhibition of carbonic anhydrase IX and XII, particularly in hypoxic invasive breast cancer, while others exhibit cytotoxicity against prostate and kidney cancer, surpassing some approved therapies [164,165,166]. Clindamycin, a well-established antibiotic derived from lincomycin, traditionally inhibits bacterial protein synthesis through binding to the 50S ribosomal subunit [167]. Interestingly, clindamycin derivatives target β-adrenergic receptors in HCC cells, while metronidazole sulfonamide derivatives inhibit EGFR and HER2 kinases in adenocarcinoma and melanoma cell lines [168,169].

Among antimycobacterial agents, isoniazid irreversibly binds monoamine oxidase A (MAOA), a mitochondrial enzyme linked to prostate cancer progression. Conjugation with near-infrared fluorescent (NIRF) heptamethine cyanine dye enhances mitochondrial targeting, inducing ROS production and apoptosis [170]. Notably, aromatic derivatives of isoniazid induce apoptosis in breast and gastric adenocarcinoma cells via caspase activation and immune modulation [171]. Additionally, pyrazinamide derivatives demonstrate activity against glioblastoma, colon, and ovarian cancers, offering insights for rational anti-cancer drug design [172].

### 10.3. Microbial-Driven Cancer Interventions

Antibiotic-based microbial interventions underscore the importance of targeting tumor-associated microorganisms. This concept has gained traction in experimental models of CRC, since *Fusobacterium*-positive CRC tumors respond significantly to metronidazole, reducing growth and progression [173]. Moreover, evidence supports that cefoxitin eliminates enterotoxigenic *Bacteroides fragilis*, mitigating IL-17a-mediated tumor development [174,175]. Additionally, antibiotics counteract the pro-cancer effects of heme-induced colonic epithelial damage and hyperplasia by eradicating sulfide-producing bacteria that degrade intestinal mucus barriers [176]. Such findings outline the potential for microbial-targeted therapies to significantly influence cancer treatment and pave the way for further exploration across various cancer types.

Overall, from β-lactam scaffolds to microbial-targeted therapies, antibiotic-based innovations hold significant promise in oncology, providing new tools to enhance efficacy and reduce toxicity. The growing understanding of these mechanisms underscores their potential to transform cancer therapy, offering a foundation for future clinical applications.

## 11. Conclusions

Since their discovery, antibiotics have been indispensable in combating infectious diseases, and their role in supporting cancer patients, who are particularly vulnerable to infections due to immune suppression and tumor progression, remains vital. Emerging insights into cancer pathophysiology, particularly the interplay between immune dysfunction, tumor progression, and microbiome dysregulation, suggest that traditional antibiotics may hold untapped potential in cancer treatment. However, despite promising experimental evidence highlighting their anti-tumor effects, most studies remain confined to preclinical models. Challenges such as conflicting data on antibiotics’ role in cancer development and concerns over antimicrobial resistance have hindered the progression to clinical trials. While concerns about the spread of antibiotic resistance and disruption of the host microbiome are valid, it is important to note that cancer presents a higher barrier of risk and toxicity due to the critical nature of the disease. Addressing these challenges requires a focused effort to unravel the precise mechanisms behind antibiotics’ anti-cancer properties. We advocate for a synergistic approach that combines antibiotics with established anti-cancer therapies to maximize therapeutic outcomes. Additionally, advances in the design of novel antibiotic derivatives and the development of targeted drug delivery systems could mitigate toxicity and resistance concerns, paving the way for the safe integration of antibiotics into cancer treatment regimens. With continued research and innovation, antibiotics may become a vital component of cancer therapy in the future.

## Figures and Tables

**Table 1:** Summary of commonly used antibiotics with anti-cancer potential: Mechanisms of action and cellular effects.

Antibiotic Type	Cell Line	Experiment Type	Mechanism of Action
Linezolid [26]	HCT-116 CRC cells	In vitro	- Enhances the anti-cancer effect of oxaliplatin - Increases growth inhibition, with a significant rise in apoptosis compared to individual drugs alone
Daptomycin [28]	MCF7 and HeLa cells	In vitro	- Selectively inhibits growth of certain cancer cell lines - Co-localizes with RPS19 in HeLa cells
Penicillin G [31]	Cervical cancer and leukemic cells	In vitro	Mitigates cell proliferation and induces cell death by downregulating MMP-11 and STAT5A
Ceftriaxone [32]	Lung cancer cells	In vitro and in vivo	- Targets Aurora B - Inhibits anchorage-independent growth - Suppresses lung tumor growth
Sulfamethoxazole (when combined with quercetin) [36]	HCT-116, HepG2, MCF-7, PC3 (colon, liver, breast, and prostate cancer cells)	In vitro and in vivo	- Induces apoptosis and cell cycle arrest since it upregulates caspase-3, and downregulates NF-κB signaling - Enhances antioxidant activity (SOD, GSH, CAT, TAC) - Protects vital organs by reducing MDA levels - Exhibits selective cytotoxicity against cancer cells
Neomycin [40]	Glioma cells	In vitro	- Inhibits cell proliferation by downregulating cyclin D1 - Arrests cell cycle by blocking p42/44 MAPK and CREB-directed transcription pathways
Neamine [43]	Oral cancer cells	In vivo	Exhibits anti-angiogenic properties by suppressing angiogenin nuclear translocation
Gentamicin [44]	Gastric cancer cells	In vitro	- Induces upregulation of aSMase and modifies sphingomyelin metabolism, leading to increased apoptosis and reduced proliferation
Erythromycin [52]	SH-SY5Y neuroblastoma cells	In vitro	- Inhibits cell proliferation in a concentration- and time-dependent manner - Leads to cell cycle arrest at S phase - Collapses mitochondrial membrane potential - Overloads cytosolic calcium - Downregulates c-Myc, and up-regulates p21 (^WAF1/Cip1^) protein expression
Clarithromycin [55]	Human CRC cells	In vitro and in vivo	- Inhibits growth by modulating autophagic flux, inducing apoptosis, and reducing Akt phosphorylation through inhibition of hERG1-PI3K interaction - Enhances the cytotoxic effect of 5-FU
Azithromycin [57]	Human GBM U87 cells	In vitro	- Reduces viability - Facilitates apoptosis - Inhibits cell proliferation
Rapamycin [68]	HNSCC cells	In vivo	Inhibits mTOR, halting malignant conversion of precancerous lesions and promoting regression of advanced carcinogen-induced SCCs
Rokitamycin [72]	Human leukemia cells	In vitro	Promotes apoptosis through DNA fragmentation, caspase activation, and mitochondrial perturbation
Doxycycline [85]	MG-63 osteosarcoma cells	In vivo and in vitro	- Activates Notch1, induces S-phase arrest, apoptosis, and autophagy in osteosarcoma cells - Blocks PI3K/Akt/mTOR signaling to mediate anti-tumor effects
Minocycline [93]	MDA-MB-435 breast cancer cells and M13SV1-Cre breast epithelial cells	In vitro	- Dampens TNF-α-induced cell fusion by targeting the NF-κB signaling pathway - Inhibits TNFR1-TRAF2 interaction, - Blocks phosphorylation of IκBα and NF-κB-p65 - Mitigates the expression of MMP-9 and ICAM1 in epithelial cells
Tigecycline [97]	Neuroblastoma cells	In vitro and in vivo	- Diminishes tumor growth and proliferation via the inhibition of the Akt pathway - Induces G1-phase cell cycle arrest - Inhibits colony formation and suppresses neuroblastoma xenograft growth
Eravacycline [101]	BxPC-3 pancreatic cancer cells	In vitro	- Inhibits cell proliferation and migration - Promotes apoptosis in PDAC cells
Ciprofloxacin [104]	SW480, SW620 (colon), PC3 (prostate), and HaCaT (normal) cells	In vitro and in vivo	- Ciprofloxacin conjugated with oleic acid induces apoptosis, reduces IL-6 release, and shows IC50 of 7.7 μM in PC3 cells (significantly lower than ciprofloxacin alone) - Conjugates also induce late apoptosis and exhibit high cytotoxicity against cancer cell lines, without affecting normal cells
Moxifloxacin [105]	HT-29 colon cancer cells	In vitro	- Enhances topoisomerase II inhibition, induces G2/M cell cycle arrest and increased apoptosis compared to etoposide alone - Reduces IL-8 and VEGF secretion, enhancing the cytotoxic effect of etoposide through increased DNA-enzyme cleavable complexes formation
Gatifloxacin [109]	Pancreatic cancer cells	In vitro	- Inhibits growth by downregulating the Akt pathway - Enhances gemcitabine sensitivity - Induces cell cycle arrest at S and G2 phases via TGF-β1-mediated p21 activation, p27 activation, and p53-dependent mechanisms, with no apoptosis observed
Metronidazole [112]	Gemcitabine-resistant cholangiocarcinoma cells	In vitro	- Enhances chemosensitivity - Mitigates cell migration - Inhibits ALDH activity, suggesting a disulfiram-like mechanism
Rifampicin [116]	Resistant leukemia and liver cancer cells	In vivo	Boosts vinblastine’s accumulation and activity by inhibiting its efflux from the cells

Abbreviations: 5-FU: 5-fluorouracil; Akt: Protein Kinase B; ALDH: Aldehyde Dehydrogenase; aSMase: Acid Sphingomyelinase; CAT: Catalase; c-Myc: MYC proto-oncogene; CRC: Colorectal Cancer; CREB: cAMP Response Element-Binding Protein; DHA: Docosahexaenoic Acid; EAC: Ehrlich Ascites Carcinoma; GBM: Glioblastoma Multiforme; GSH: Glutathione; HCT-116: Colorectal Cancer Cells; HeLa: Cervical Cancer Cells; hERG1: Human ether-à-go-go-related gene 1; HNSCC: Head and Neck Squamous Cell Carcinoma; IC50: Half maximal inhibitory concentration; IκBα: Inhibitor of nuclear factor kappa-B kinase alpha; IL-6: Interleukin-6; LC3: Microtubule-associated Protein 1A/1B-light Chain 3; MAPK: Mitogen-activated protein kinase; MDA-MB-435: Human Breast Cancer Cells; MMP: Matrix Metalloproteinase; Moxifloxacin: A Fluoroquinolone Antibiotic; mTOR: Mechanistic target of rapamycin; NF-κB: Nuclear Factor Kappa B; Notch: Νeurogenic locus notch homolog protein; PC3: Prostate Cancer Cells; PDAC: Pancreatic Ductal Adenocarcinoma; PI3K: Phosphoinositide 3-Kinase; p21: Cyclin-Dependent Kinase Inhibitor 1A; p27: Cyclin-dependent Kinase Inhibitor 1B; p53: Tumor Protein 53; p62: Sequestosome-1; RPS19: Ribosomal Protein S19; SOD: Superoxide Dismutase; STAT5A: Signal Transducer and Activator of Transcription 5A; SW480: Colorectal Cancer Cells; SW620: Metastatic Colorectal Cancer Cells; TAC: Total Antioxidant Capacity; TGF-β1: Transforming Growth Factor Beta 1; TNF-α: Tumor Necrosis Factor Alpha; TNFR1: Tumor necrosis factor receptor 1; TRAF2: TNF receptor-associated factor 2; VEGF: Vascular Endothelial Growth Factor.

## Data Availability

Not applicable.

## Abbreviations

5-FU: 5-fluorouracil; ADAMTS1: ADAM metallopeptidase with thrombospondin type 1 motif 1; AI: Artificial Intelligence; Akt: Protein kinase B; ALDH: Aldehyde dehydrogenase; AMPK: AMP-activated protein kinase; aSMase: Acid Sphingomyelinase; BAX: B-cell lymphoma 2-associated X protein; Bcl-2: B-cell lymphoma 2; Bcl-xL: B-cell lymphoma-extra-large; Bmi-1: B cell-specific Moloney murine leukemia virus integration site 1; CA-4: combretastatin A-4; Casp: Caspase; CAT: Catalase; CD133: prominin-1; CDK: Cyclin-dependent kinase; CIK: Cytokine-induced killer cells; CLA: Clarithromycin; CML: chronic myeloid leukemia; C-PARP1: Cleaved poly (ADP-ribose) polymerase 1; CRC: Colorectal cancer; CREB: cAMP response element-binding protein; CRISPR-Cas: Clustered regularly interspersed short palindromic repeats-CRISPR associated protein; CSC: Cancer stem cells; CXCR4: C-X-C chemokine receptor 4; DDIT3: DNA damage inducible transcript 3; DENA: Diethyl-nitrosamine; DOX: Doxycycline; EAC: Ehrlich ascites carcinoma; EGF: Epidermal growth factor; EGFR: Epidermal growth factor receptor; EMT: Epithelial-to-mesenchymal transition; ER: Endoplasmic reticulum; ERK: Extracellular signal-regulated kinase; ERV: Eravacycline; FAs: Fatty acids; FZD10: frizzled class receptor 10; GADD45A: growth arrest and DNA damage inducible alpha; GB: glioblastoma; G-CSF: Granulocyte colony-stimulating factor; GSH: Glutathione; HCC: hepatocellular carcinoma; hERG1: Human ether-à-go-go-related gene 1; HER2: Human epidermal growth factor receptor 2; HMOX1: heme oxygenase 1 gene; hNanog: homeobox protein NANOG; HR-negative hormone receptor-negative; Hsp90: heat shock protein 90; HTS: high-throughput screening; HUVE: Human umbilical vein endothelial; IBD: inflammatory bowel disease; IC50: Half maximal inhibitory concentration; ICAM-1: Intercellular adhesion molecule-1; ICIs: Immune checkpoint inhibitors; IFN-α: interferon-alpha; IFN-γ: interferon-gamma; IκBα: Inhibitor of nuclear factor kappa-B kinase alpha; IL: Interleukin; IRN: Irinotecan; IRF-1: Interferon regulatory factor-1; JNK: c-Jun N-terminal kinase; JUN: Jun proto-oncogene; Kₑ: dissociation constant; LC3-II: Microtubule-associated protein 1A/1B-light chain 3-II; LC3B-II: microtubule-associated protein 1A/1B-light chain 3B type II; LDH: Lactate dehydrogenase; MAOA: Monoamine oxidase A; MAPK: Mitogen-activated protein kinase; MDA: Malondialdehyde; MDR: Multidrug-resistant; MIN: Minocycline; MITF: microphthalmia-associated transcription factor; MMP: Matrix metalloproteinase; MOX: Moxifloxacin; mTOR: Mechanistic target of rapamycin; MTA: microtubule-targeting agent; MUC1: Mucin 1; NF-κB: Nuclear factor kappa-light-chain-enhancer of activated B cells; NIRF: near-infrared fluorescent; NK: Natural killer; NLRP3: NOD-like receptor P3; Notch: neurogenic locus notch homolog protein; NSCLC: Non-small-cell lung cancer; OCT-4: Octamer-binding transcription factor 4; OS: overall survival; OXPHOS: Oxidative phosphorylation; p70S6K: p70 ribosomal protein S6 kinase; PARP: Poly (ADP-Ribose) polymerase; PD-1: Programmed cell death protein 1; PD-L1: Programmed cell death ligand 1; PFS: Progression-free survival; PI3K: Phosphoinositide 3-kinase; PRISM: Profiling relative inhibition simultaneously in mixtures, PSA: Prostate-specific antigen; QCT: Quercetin; QoL: Quality of Life; RAF1: Rapidly accelerated fibrosarcoma 1; ROS: Reactive oxygen species; RPS19: Ribosomal Protein S19; SCFAs: Short-chain fatty acids; SCID: Severe Combined Immunodeficiency; SMX: Sulfamethoxazole; SOD: Superoxide dismutase; SOX2: SRY-box transcription factor 2; STAT5A: Signal transducer and activator of transcription 5A; TIG: Tigecycline; TME: Tumor microenvironment; TMP-SMX: Trimethoprim-sulfamethoxazole; TNBC: triple-negative breast cancer; TNF-α: Tumor necrosis factor-alpha; TNFR1: Tumor necrosis factor receptor 1; TRAF2: TNF receptor-associated factor 2; VEGF: Vascular endothelial growth factor.
